# Alzheimer’s disease detection using a quantum deep neural network with Haralick feature extraction and simulated annealing optimization

**DOI:** 10.7717/peerj-cs.3387

**Published:** 2026-02-10

**Authors:** Sabari Vasan S., Jayalakshmi P.

**Affiliations:** School of Computer Science Engineering and Information Systems, Vellore Institute of Technology University, Vellore, Tamil Nadu, India

**Keywords:** Alzheimer disease, Quantum computing, Simulated annealing, Haralick feature extraction, Deep neural network

## Abstract

Alzheimer’s disease (AD) is a neurodegenerative disorder that affects a wide range of individuals worldwide. It is of utmost importance to detect AD at an earlier stage and diagnose it to manage the disease effectively. Detecting AD using traditional methodologies is not cost-effective and time-consuming because of the clinical tests and neuroimaging methods involved. Over the last few years, quantum computing and deep learning (DL) have become practical approaches for detecting and diagnosing AD. Unlike conventional methods, quantum computing allows for faster solving complex and entangled computable problems. DL models have a high potential for automatically learning and extracting pertinent features even from larger datasets. Hence, a new approach combining multiple concepts such as deep neural network (DNN), quantum computing, simulated annealing (SA) optimisation, and Haralick feature extraction has been proposed in this work for detecting AD. A quantum deep neural network (QDNN) is introduced in this article to take over the extraordinary computational capability of quantum systems. Haralick feature extraction is implemented in this study to extract the texture features from the medical images, resulting in a rich feature set for the model. The dataset used in this study, The Best Alzheimer’s MRI Dataset contains 11,519 axial MRI images in .jpg format with a resolution of 128 × 128 pixels, categorised into four balanced classes—no impairment, very mild impairment, mild impairment, and moderate impairment—each comprising 2,560 images. To optimise the Haralick features from medical images and to enhance the model’s learning process with optimised parameters, a new feature-specific simulated annealing method (FSSA) has been introduced in this article. The experimental results proved that our model achieved an accuracy of 98%, a precision of 99%, a sensitivity of 97%, and a specificity of 98%. The results achieved in this study are better than the traditional model’s performance, and thus better in all performance metrics. The results indicated that the proposed QDNN model is a good framework for AD detection.

## Introduction

Alzheimer’s disease (AD) is a brain disorder that affects the memory, behaviour, and thoughts of the person diagnosed with it. Although it can be identified with the decline in the mental abilities of the affected person, accurate identification of AD is necessary for making the treatment and patient care more successful. Many technological advancements have been recently made, especially in deep learning (DL) and machine learning (ML), thus making AD diagnosis easier using these techniques (Kavitha et al., 2022). Similarly, these algorithms are leveraging techniques in selecting and extracting the features, thus enabling the identification of subtle patterns that indicate AD in its earlier stage (Shukla, Tiwari & Tiwari, 2023; Jahan et al., 2023; Gu et al., 2022; Kim, 2023). Researchers have implemented many methodologies, including various DL models, feature selection and extraction techniques, and improvised classification algorithms for analysing the patterns and anomalies in MRI images associated with AD (Liu et al., 2022). These methodologies aim to find any subtle alterations in the brain structure in the early stages of AD, particularly when the intervention strategies are more effective. Deep neural network (DNN), when combined with quantum computing, has exhibited promising results in medical diagnostics (Boo et al., 2021; Zhao & Gao, 2021).

Similarly, texture-based feature extraction methods obtained from grey-level co-occurrence matrix (GLCM), such as Haralick features, have been proven effective in capturing structural and textural abnormalities in medical imaging datasets (Haralick, Shanmugam & Dinstein, 1973). However, integrating these approaches with advanced optimisation techniques has not been significantly explored in the context of AD diagnosis. Haralick feature extraction is gaining popularity because it can effectively capture texture information from medical images. In addition, it plays an irreplaceable role in improving the discriminative power of the model. Henceforth, identifying diagnostic complex designs and textures is made easier in the evolution of AD. Often, there are limitations to the conventional methods available for AD detection in terms of precision and early-stage identification. With the implementation of quantum physics, quantum computing differs from traditional computing regarding information processing (De Britto, 2024). Integrating quantum principles into DNNs will allow the management of complicated data more effectively. Quantum deep learning (QDL) is an invention that integrates the representational strengths of deep learning and the computational strengths of quantum computers providing an innovative solution to traditional deep learning (Azevedo, Silva & Dutra, 2022; Garg & Ramakrishnan, 2020). Although the traditional DL models are highly effective, they have shortcomings in terms of scalability when handling large and complex datasets as classical computing resources are limited (Garg & Ramakrishnan, 2020; Kumar & Tripathi, 2019). QDL uses quantum parallelism and superposition to allow a large number of data processing in parallel, giving it faster convergence and better performance over high-dimensional and entangled feature spaces, enabling high-dimensional feature spaces frequently found in medical imaging tasks such as detecting Alzheimer’s disease. In addition, quantum algorithms have the potential to search complex optimization problem space in a more effective way and thereby avoid local minima more easily, and hence better generalization of the model. These advantages render QDL especially appealing as a method of extracting complex patterns in noisy or high-resolution biomedical data, with relatively greater accuracy, reduced training overhead, and improved management of computationally expensive feature interactions than conventional DL algorithms. During the last few years, simulated annealing (SA) has been growing owing to its ability to solve optimisation problems, which is called a metallurgical annealing approach (Hajji, Hamlaoui & Hadda, 2024; Matear, 1995). As this can escape from the local optimum solution, it helps optimise the feature selection and weighing process even in large datasets. Recent studies have suggested using SA and machine learning methodologies to enhance classification accuracy and the computational speed of medical imaging applications (El Amoury, Smili & Fakhri, 2025).

Despite such advances, the application of SA to optimise feature-specific weights, particularly in texture-based Haralick features, for detecting AD remains an area to be thoroughly explored. The method also optimises the model parameters. Optimisation techniques such as these guarantee an optimal global solution and a globally robust and accurate AD detection system. An effective solution to AD diagnosis is presented in this article, which integrates feature-specific simulated annealing method (FSSA) with Haralick feature extraction and QDNN. SA optimises the Haralick features to emphasise the most critical texture patterns for diagnosis, whereas QDNN employs quantum-inspired methods to enhance learning.

The main contributions of this study are as follows.

The integration of QDNN offers a novel approach to AD detection, leveraging the unique computational principles of quantum computing.

Haralick feature extraction is employed to capture intricate textural information from medical images. Additionally, we aimed to enhance the model’s potential capability to identify small patterns predictive of AD progression.

To propose a new FSSA to optimise the Haralick features extracted from medical images and use the optimised features in a QDNN for improved AD diagnosis performance. It also optimises the parameters of the QDNN model to achieve an optimal solution from a global perspective.

The organisation of the task is described as follows. A detailed discussion of the current techniques and tools used for detecting AD is presented in “Related Works”. Following this, the theoretical foundations and technical details required for a better understanding of the proposed methodology are given in “Methodology”. The results of the proposed study are presented in “Experimental Result and Discussion”. Finally, the conclusion of the proposed work is detailed in “Conclusion”.

### Related works

The importance of plasma biomarkers in anticipating cognitive decay in older people without cognitive impairment was highlighted by Cullen et al. (2021), particularly in association with AD. Kim et al. (2021) demonstrated a DL-based algorithm by using the 2-[18F] FDG-PET imaging data to categorise amyloid PET-positive. Chang, Lin & Lane (2021) highlighted the use of ML and new biomarkers for AD detection to improve specificity and sensitivity. Following this analysis, the authors suggested that implementing ML with various biomarkers will improve AD diagnostic accuracy (Cano et al., 2021). Other research in the field has also supported integrating new technologies and biomarkers for detecting AD at an earlier stage (Winchester et al., 2023).

Abate et al. (2020) introduced a p53 conformation variation and ML for earlier diagnosis of AD in preclinical and prodromal phases. A DL model has been introduced by Qiu et al. (2020) for AD classification with the help of MRI techniques. While generating NMDA receptor antagonists that are highly potent, Schultz et al. (2021) explored DL implementation. A variational autoencoder (VAE)-based model has been utilised to explore the chemical space, thereby developing potential chemical structures for NMDA receptor antagonists. ML was employed by Popuri et al. (2020) to assess AD neurodegeneration patterns *via* structural MRI. de Mendonça & Ferrari (2023) demonstrated a study in which AD classification was carried out. According to Ingala et al. (2022), quantitative atrophy assessments using MRI are clinically beneficial for evaluating patients susceptible to AD. Prescott (2022) used the Dominantly Inherited Alzheimer Network (DIAN) cohort to examine the connection between PET amyloid load and diffusion tensor MRI connectivity to structures in preclinical autosomal dominant AD. Hybrid DL models were proposed by Abuhmed, El-Sappagh & Alonso (2021) to look for signs of AD. El-Sappagh et al. (2021) used the early integration of low-cost multimodal data with both comorbidity and medication features for the detection of AD. SA was utilised by Qureshi et al. (2022) to overcome the inverse problem of limited-view computed tomography (CT). JiMing et al. (2024) proposed a unique hybrid algorithm, SAOBL-IA, which combines SA, opposition-based learning (OBL), and immune algorithm (IA). A hybrid whale optimisation algorithm was implemented along with Whale Optimization Algorithm with Simulated Annealing (WOA-SA) in a study demonstrated by Rajathi & Jiji (2019). This was implemented to accurately classify various types of chronic liver disease. In addition, WOA-SA was used to select the optimal features required to fine-tune the chronic liver disease (CLD) classification accuracy for five types of liver diseases.

Jenber Belay, Walle & Haile (2024) proposed an ensemble learning model based on the idea to use quantum machine learning classifiers along with AD. In their system, the merged images were taken as the input and their salient aspects were extracted with tailored settings of VGG16 and Resnet50 where the same were applied to the quantum classifier as a final step to make predictions. Equally, Shahwar et al. (2022) established quantum-classical hybrid architecture by using a quantum transfer variational model that increased the test accuracy of the AD model by enabling faster identification of the disease. A similar study was conducted by Sercek et al. (2025), who also suggested a feature engineering method that combined a quantum-inspired generalized histogram of patterns (GHP) feature extraction with a self-organized architecture to identify AD based on EEG signals, reaching a classification accuracy of 88.17. Table 1 is a comparative summary of other existing methods.

**Table 1:** Comparison of different methods.

Author	Method	Dataset	Advantage	Disadvantage
Baglat et al. (2022)	Multiple ML models	OASIS dataset	Achieved accuracy rate of 86%.	The lack of complex AI-based methods used in the current study can be a limitation of its predictive ability as compared to the possibilities of DL and hybrid methods application.
Ching, Abdullah & Shapiai (2024)	EfficientNet-B0	MRI scans	With an accuracy of 87.17%.	Limitation of the model entails that it does not perform well with complex features representation.
Alsubaie, Luo & Shaukat (2024)	CapsNets	Brain image	It is beneficial in minimising overfitting.	It is computationally intensive and takes longer to train than normal CNNs. They are also not very scalable and it is challenging to process large scale datasets.
Raza et al. (2023)	DenseNet-169 and ResNet-50	OASIS and openly available MRI scans	It extracts complex patterns of neuroimaging information.	These models have the disadvantage of being influenced by performance deterioration when processing unbalanced datasets.

### Methodology

**Figure 1:**
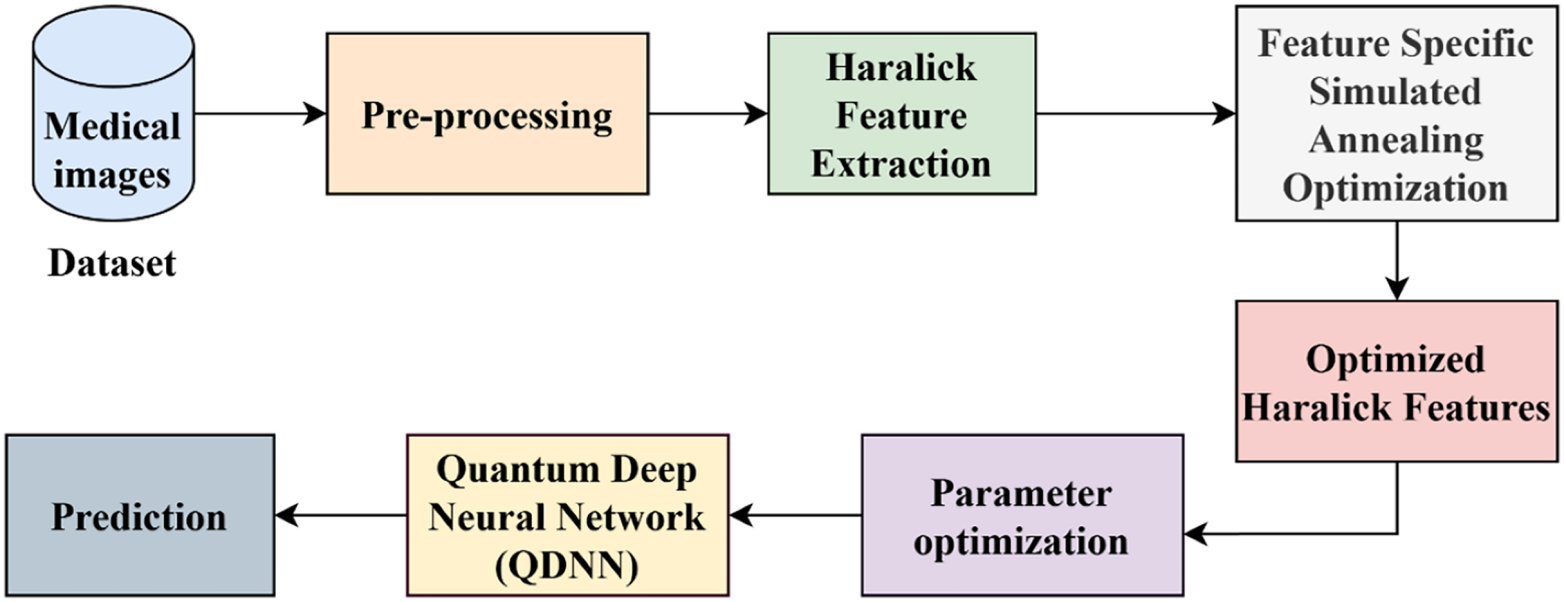
Proposed workflow.

The proposed model architecture of AD detection combines traditional feature engineering, optimisation, and quantum deep learning. First, preprocessing of medical image datasets is performed to improve their quality and make them ready to analysis. The Haralick feature extraction is, then, applied to identify fundamental texture patterns that are imperative in differentiating AD-related changes. These features obtained are then optimised by the feature-specific simulated annealing (FSSA) method to enhance feature relevance and minimise redundancy. The optimised features are then compiled into a quantum deep neural network (QDNN) where parameters are optimised to reduce the AD detection loss function. The architecture enables us to couple the computational power of quantum computing to the capabilities of classical neural networks and feature optimisation, resulting in a robust and accurate framework capable of automated AD prognosis, which has potential to lead to early diagnosis and interception. The full workflow of the suggested approach is shown in Fig. 1.

### Dataset

The Best Alzheimer MRI Dataset of (99% Accuracy) (Chugh, 2025) collection by Luke Chugh has 11,519 motionless MRIs in .jpg format and measures 128 × 128 pixels. The images are divided into four classes: No impairment, very mild impairment, mild impairment, and moderate impairment, which are comprised of 2,560 images each. The data is properly balanced, optimised to detect and classify Alzheimer’s disease, with a total size of around 75 MB, which is appropriate to perform ML-based studies in neuroscience.

### Data preprocessing

Datasets, especially in MRI analysis, exhibit irregularities such as unusual data combinations, missing values, and redundant information. To guarantee the accuracy of subsequent evaluations, these problems must be resolved. Regarding the Kaggle dataset, further processing using FreeSurfer was performed to enhance the data quality. The preprocessing steps encompassed several key procedures.

(a) Handling missing values: Addressing missing values is essential to avoid bias and errors in subsequent analyses.
(1) $$Mean\; Imputation\! :{\rm xi} = \sum \limits_{j = 1}^n {x_j},$$
${x_j}$: The missing value to be imputed, $\sum n$: The sum of all available values, and $n$: $j = 1.$
  The number of available values. Mean imputation replaces the missing values with the mean of the available non-null values. This will provide a simple and effective approach.

(b) Head Motion Correction: Head motion during MRI scans can introduce artefacts. To mitigate these effects, head motion correction techniques, often involving image registration algorithms, have been applied.
(2) $$Icorrected = MotionCorrection\left( {Iraw} \right).$$

(c) Compensation for Slice-Dependent Time Shift: Compensation techniques measure the variations in acquisition time between the slices.
(3) $$I_{compensated} = TimeShiftCompensation\left( {I_{corrected}} \right).$$
  Smoothing: Smoothing algorithms like Gaussian smoothing are applied to reduce the noise in the images.
(4) $$Ismoothed = Gaussiansmoothing\left( {Icompensated} \right).$$

(d) Normalisation: Normalisation is performed to ensure consistent scale and facilitate meaningful comparisons.
(5) $$Inormalized = Intensitynormalization\left( {Ismoothed} \right).$$

(e) Normalisation of Brain Volume Measurements: Brain volume measurements, including various regions were normalised by the intracranial volume (ICV) to account for individual head sizes.
(6) $$NormalizedMeasurement = \displaystyle{{Raw\; measurement} \over {ICV}}.$$
  By meticulously addressing these preprocessing steps, we enhanced the quality of the MRI data, ensuring more accurate and reliable analyses.

### Haralick feature extraction

Using the Kaggle dataset, Haralick feature extraction can yield useful information on the textural properties of brain images, which can then be utilised as input features for ML models in identifying AD. Haralick feature extraction, a concept based on GLCM, provides information about texture patterns existing in a picture by capturing spatial relationships between pixel pairs. The technique can be applied especially to detecting Alzheimer’s as a result of neurodegeneration, which can cause minor changes in structure and texture of brain tissue that can still be measured through analysis of texture values without it being visibly noticeable. Pixel pairings with particular intensity values and spatial relationships within a designated neighbourhood were counted to create the GLCM. Features can be extracted from brain images using the following methods: before performing Haralick feature extraction, it is important to preprocess the brain images to enhance their quality and remove artefacts or noise.

GLCM Calculation: Once brain images are preprocessed, GLCMs can be calculated. For each pixel in the image, the GLCM is constructed by considering its neighbouring pixels within a defined distance and angle. The GLCM captures the frequency of various pixel pair occurrences with specific intensity values and spatial relationships. Haralick Feature Calculation: A set of Haralick features can be calculated from the GLCMs. These features evaluate several elements of texture, such as contrast, correlation, energy, and homogeneity. Haralick features’ calculation involves applying mathematical formulas to the GLCMs.

GLCM: Assume $P\left( {i,j} \right)$ represents the probability of occurrence of pixel intensity values $i$ and $j$ at a given spatial relationship. The GLCM is defined as:

(7) $$GLCM\; \left( {d,\theta } \right) = \left[ {P\left( {0,0} \right)P\left( {0,1} \right) \ldots P\left( {255,255} \right)} \right],$$

where $d$ denotes the distance between pixel pairs and $\theta$ represents the direction. The more comprehensible picture of the texture structure is obtained by calculating GLCMs in the contrasting directions and distances. The commonly used Haralick features measuring the variations in local intensity in the image can be calculated as shown in Eq. (8):

(8) $${\rm C}ontrast = \sum \limits_{i = 0} 255\mathop \sum \limits_{j = 0} 255{\left( {i - j} \right)^2}.P\left( {i,j} \right),$$

where symbols $i$ and $j$ stand for the intensity values of the two pixels, the normalised GLCM value is denoted by $P\left( {i,j} \right)$ for the pixel pair $\left( {i,j} \right)$. Gray level contrasts are higher in the case of local differences in intensity and this could be related to structural deformities attributable to AD pathology.

Energy, an important Haralick feature representing uniformity or homogeneity of texture, can be calculated as a sum of squared GLCM values, as shown in the following Eq. (9):

(9) $${\rm Energy} = \sum \limits_{i = 0} 255\mathop \sum \limits_{j = 0} 255{\left( {P\left( {i - j} \right)} \right)^2}.$$

Transitions in areas with highly homogeneous texture shall give higher energy values, whereas regions of heterogeneity run rampant in diseased tissues shall give lowered energy values. Similarly, other Haralick features, such as homogeneity, correlation, and entropy, can be calculated using the following Eqs. (10), (11) and (12):

(10) $${\rm Correlation} = \displaystyle{{\sum \nolimits_{{i} = 0}^{255} \sum \nolimits_{{j} = 0}^{255} \left( {{i}.{j}.{P}\left( {{i},{j}} \right)} \right) - {{\mu }_{x}}.{{\mu }_{y}}} \over {{{\sigma }_{x}}.{{\sigma }_{y}}}}$$

(11) $$Homogeneity = \sum \limits_{i = 0}^{255} \sum \limits_{j = 0}^{255} \displaystyle{{P\left( {i,j} \right)} \over {1 + \left| {i - j} \right|}}$$

(12) $$Entropy = \sum \limits_{i = 0}^{255} \sum \limits_{j = 0}^{255} P\left( {i,j} \right).\log_{2}\left( {P\left( {i,j} \right) + \epsilon } \right),$$

where ${{\mu }_{x}}$, ${{\mu }_{y}}$, ${{\sigma }_{x}}$ and ${{\sigma }_{y}}$ in the above equation represent the mean and standard deviation of the pixel intensities. Correlation consists of measuring the linear dependence between pixel pairs, homogeneity consists of examining the closeness of the distribution of elements to the GLCM diagonal, and entropy measures the randomness of texture but usually more so in regions of diseased tissue.

Obtaining such set of Haralick features of the MRI scans provides the proposed model with a rich and quantifiable set of texture descriptors that can lead to an increased discrimination between the stages of Alzheimer.

**Algorithm 1:** Pseudocode for Alzheimer’s disease detection using Haralick features.

Input: Kaggle Image dataset
Output: Selected Haralick feature
Begin
Preprocess each image in the dataset.
Calculate the Grey-Level Co-occurrence Matrix (GLCM) for each image.
Extract Haralick features such as Contrast, Energy, Correlation, Homogeneity, and Entropy from the GLCM.
Perform feature selection to identify the most discriminative and informative features.
Return the selected Haralick features.
End

**Algorithm 2:** Training and prediction of Alzheimer’s disease using QDNN model.

Input: Kaggle Image dataset
Output: Predicted labels for Alzheimer’s Disease
Begin
Extract Haralick features from each image in the Kaggle dataset
Store the extracted Haralick features.
Select the most relevant features from the stored Haralick features.
Train a QDNN (Quantum Deep Neural Network) model using the selected features and labels.
Store the trained model.
Use the trained model to predict Alzheimer’s Disease (AD) based on test features.
Store the predicted labels.
Return the predicted labels.
End

### Feature-specific simulated annealing

The detection of AD using medical imaging entails feature extraction from brain scans of a broad range of features, such as texture, shape, and intensity patterns. Of these, Haralick features derived from GLCM were found to be useful in delineating detailed textural information. These attributes pick up fine differences in brain tissue composition that may not be visible to the naked eye, yet can be a marker of early neurodegeneration. Not all features have equal significance in the diagnosis process; some can introduce redundancy and noise. Excessive features do not only introduce irrelevant computational overhead, but can also obscure the model with irrelevant patterns. Optimisation of feature selection and weights is essential to ensure that only the most important features are used in the diagnosis, decreasing computational complexity and improving model performance. The FSSA meets this requirement by concentrating on optimising the individual relevance of every feature, instead of treating them as equals. In contrast to standard feature selection methods, which completely eliminate less significant features, FSSA re-weights each feature, allowing the model to keep potentially valuable minor patterns, but stack their effect. It presents a new, focused optimisation paradigm for Alzheimer’s detection through dynamically weighting the Haralick features. This method enhances diagnostic accuracy and computer efficiency by identifying and optimising significant features in the process of disease diagnosis.

Initially, random weights are allocated to each extracted feature. Let us assume weights ${w_x}$ allocated to every feature ${f_x}$, such that ${w_x} \in \left[ {0,1} \right]$.

The weighted feature vector may be written as,

(13) $${F_w} = \left\{ {{w_1}{f_1},{w_2}{f_2},{w_3}{f_3}, \ldots ..{w_n}{f_n}} \right\}.$$

In the above equation, the weight for the $n$th feature can be denoted as ${w_n}$, and $n$ denotes the overall number of features.

The objective function, or $obj$, can be formulated to assess the quality of the weighted features and their contribution to the QDNN model.

(14) $$Obj\; = a.accuracy - b.redundancy,$$

where $a$ and $b$ are hyperparameters for balancing the objectives. The accuracy is calculated here by the classification performance of the QDNN on the validation set, and redundancy is determined by the mean pairwise correlation within features, where high correlations between features are penalised.

SA adjusts the feature weights $w$ to maximise the objective function.

*Initialisation process:* The optimisation process employs an initial temperature of ${T_0}$, a cooling schedule of $T\left( t \right),$ and initial feature weights of ${w_0}$. The initial temperature ${T_0}$, governs the likelihood to accept poorer solutions at early stages of the search to avoid local minima, and the cooling pattern lowers this likelihood progressively.

*Perturbation:* By perturbing the existing solution $w$, a new candidate solution $w'$ can be generated as:

(15) $$w_n^{\prime} = \; {w_n} + \delta.$$

In Eq. (15), Gaussian noise with zero mean can be denoted as $\delta$ and variance can be denoted as ${\sigma ^2}$. This random perturbation brings the element of diversity in the search process that enables the algorithm to search a wide solution space.

*Acceptance criteria:* The change in the objective function $(ob{j^{\prime}})$ can be calculated, and then the new solution with probability can be accepted.

(16) $$P = \left\{ {\matrix{1, & obj^{\prime} > 0 \cr e^{{obj^{\prime}} \over T}, & obj^{\prime} \le 0 \cr } } \right. .$$

This probabilistic acceptance rule implies that an inferior solution can be accepted at an early stage in order not to be trapped in local optima and, the lower $T$, the less probable the solution will be accepted.

*Cooling Schedule:* Reduce the temperature $T$ using a predetermined cooling plan that includes exponential decay.

(17) $$T\left( {t + 1} \right) = T\left( t \right).\gamma.$$

In the above equation, $\gamma$ ranges from 0 to 1. Lower $\gamma$ causes faster cooling, which can lead to premature convergence, and higher values near 1 cause slower cooling and more exploration.

*Termination step:* The optimisation ends if $T$ is less than a value or after a specified number of iterations. Upon termination, the resulting set of weighted features is an optimal combination of accuracy and minimum redundancy. With this weighted feature set, the QDNN enhanced the model’s performance.

### QDNN

Utilising quantum bits, or qubits, a quantum neural network makes use of the concepts of quantum technology to enable more reliable and effective calculations. The architecture of a QDNN involves quantum gates, layers, and classical neurons, making it a hybrid model that combines quantum and classical computing elements. The QDNN incorporates quantum computing principles into traditional DNNs, allowing for the exploitation of quantum superposition and entanglement. Using these concepts, QDNNs are able to analyze high-dimensional data spaces in parallel, effectively allowing them to mine complex patterns and relations that more traditional methods of neural networks may not reach. This intrinsic parallelism greatly speeds up the process and lowers the computational overhead of nondeterministic polynomial (NP)-hard problems in the classical context.

This can more efficiently handle complex computations involved in analysing medical datasets, such as Kaggle. Setting the initial settings entails specifying its fundamental elements, including the number of qubits in each layer, quantum gates, and classical neurons. The quantum parallelism and expressive capability of a QDNN are determined by the number of qubits in each layer. The hidden layers contained M qubits, the output layer contained K qubits, and the input layer contained $N$ qubits. The entire qubit count is the result of $N\; + \; M\; + \; K$.

(18) $$Totalnumberofqubits = N + M + \; K.$$

### Steps of Alzheimer’s disease detection

(1) Initialise quantum and classical neural network parameters.
(2) Encode input data into quantum states.
(3) Define the QDNN architecture incorporating quantum gates.
(4) Apply SA for optimising quantum gate parameters.
(5) Train the QDNN using the Kaggle dataset with labelled samples.
(6) Evaluate the trained model on a separate test dataset.
(7) Extract quantum features and perform disease prediction.
(8) Optimise model performance using SA iterations.
(9) Repeat steps 4–8 until convergence or desired performance is achieved.

### QDNN structure

The initialisation process involves defining the characteristics of each layer. In a QDNN, it is a vital step since in comparison with classical neural networks, where weighted connections and bias are defined, here qubit states preparation and entanglement schemes are the key factors defining how the model will process the data provided as well as memorize it as the learning progresses. The architecture consists of the input layer, hidden layers, and output layer that are referring to the particular quantum states and gate operations.

(a) Input Layer Initialisation: The input layer of QDNN corresponds to the features extracted from the Kaggle dataset. Let $|{\psi _{input}}\rangle$ represent the quantum state of the input layer. $N$ represents No. of the qubits in the input layer, the input state is prepared as follows:
(19) $$|{\psi _{input}}\rangle = \displaystyle{1 \over {\sqrt {{2^N}} }}\mathop \sum \limits_{i = 0}^{{2^N} - 1} {\rm \mid }i\rangle .$$
  This terminology is used to describe a uniform superposition state in all computational basis states of $N$ qubits, often used as an initial point in quantum computing since multiple states can be processed in parallel. This allows the QDNN to accelerate the process of evaluating multiple combinations of the features of the dataset by encoding these features into this superposition, and then leveraging quantum parallelism.

(b) Hidden Layer Initialisation: The quantum computation takes place in the hidden layer.
  Let $|{\psi _{hidden}}\rangle$ represent the quantum state of the hidden layer. $M$ represents the number of qubits present in the hidden layer, and initialisation involves applying Hadamard gates $\left( H \right)$ to each qubit.
(20) $$| {{\psi _{hidden}}\rangle = \displaystyle{1 \over {\sqrt {{2^M}} }}\mathop \sum \limits_{j = 0}^{{2^M} - 1} |j\rangle = {H^{ \otimes {M}}}} |{\psi _{hidden,0}}\rangle .$$
  Here, ${H^{ \otimes {M}}}$ denotes the tensor product of $M$ Hadamard gates, and $|{\psi _{hidden,0}}\rangle$ is the initial state of the hidden layer. Hadamard gates are used to put every qubit in the hidden layer into an equivalent superposition, so that the QDNN can search through all possible state configurations simultaneously. This step serves a similar purpose to weight initialisation in classical networks, although in the quantum case, it also allows the future possibility of quantum entanglement and interference phenomena in subsequent calculations.

(c) Output layer initialisation: The output layer is a single qubit that provides the final prediction.
  Let $| \psi_{output} \rangle$ represent the quantum state of the output layer. The initialisation involves setting
  the output qubit to the output qubit to the ${\mid }0\rangle$ state.
(21) $$|\psi_{output} \rangle = |0\rangle .$$
  Initializing the qubit in the $|0\rangle$ state establishes a reference point of the output qubit, prior to applying quantum operations and measurements. This is essential since transformations that are subsequently introduced to the circuit will solely affect the likelihood of obtaining the output potential state $|1\rangle$ or $|0\rangle$ upon measurement, which is the outcome of classification in AD detection.

(d) Quantum Feature Encoding: Consider each sample’s dataset with $D$ features. The goal maps these features into a quantum state that can be represented on qubits.
  For a given feature ${x_i}$ from the dataset, the amplitude encoding is expressed
(22) $$|{x_i}\rangle = \sqrt {P\left( {{x_i}} \right)} |0\rangle + \sqrt {1 - P\left( {{x_i}} \right)} |0\rangle .$$

Here, $P\left( {{x_i}} \right)$ represents the probability amplitude associated with the basis state $|0\rangle$ based on the feature value ${x_i}$.

Let us consider a simplified case with two features, ${x_1}$ and ${x_2}$, for a single sample:

(23) $$|{x_1}\rangle = \sqrt {P\left( {{x_1}} \right)} |0\rangle + \sqrt {1 - P\left( {{x_1}} \right)} |1\rangle$$

(24) $$ |{x_2}\rangle = \sqrt {P\left( {{x_2}} \right)} |0\rangle + \sqrt {1 - P\left( {{x_2}} \right)} |1\rangle.$$

The quantum state of the sample is

(25) $$|X\rangle = |{x_1}\rangle \otimes |{x_2}\rangle .$$

This encoding of the MRI-derived information in quantum state amplitudes allows the QDNN to compactly represent the large and complex medical records. This procedure guarantees that every classical feature has an observable effect on the quantum state that allows high-level transformations in the next layers to recognize the pattern.

This layered initialisation and feature encoding model will guarantee that the superposition, entanglement, and interference quantum principles can be completely exploited by the QDNN to achieve higher predictive accuracy.

### QDNN forward pass

(a) Quantum state propagation: The quantum state transition from the input layer to the hidden layer can be demonstrated using the following equation:
(26) $$|{h_j}\rangle = \sum \limits_{i = 1}^N {W_{ji}}|{x_i}\rangle .$$
  Here, the symbol ${W_{ji}}$ represents the connections from the $i$th qubit of the input layer’s $j$th qubit to the hidden layer. During this phase, the quantum parallelism property can be used to concurrently process many input states, potentially resulting in large computation speedups relative to classical neural networks when applied to high-dimensional data, *e.g*., MRI scans.
(b) Activation function: Applying an activation function $f\;$ to introduce nonlinearity.
(27) $$|{{a}_{j}}\rangle = {f}\left( {|{{h}_{j}}\rangle } \right) .$$
  This introduces the classical aspect of quantum computation, bridging the quantum and classical domains.
(c) Quantum to Classical Transition: Quantum information is translated into a classical form by measuring the qubits in the hidden layer:
(28) $$|{a_j}\rangle = measure\left( {|{a_j}\rangle } \right).$$
  The measured classical values ${a_j}$ serve as inputs to the output layer.
(d) Output Computation: The quantum state $|0\rangle$ in the output layer is determined by a weighted sum of the measured classical values:
(29) $$|o\rangle = \sum \limits_{j = 1}^M {V_j}|{a_j}\rangle .$$
  Here, ${V_j}$ represents the weights that connect the output and hidden layers.
(e) Final activation function: A final activation function ${\rm g}$ is applied to obtain the output prediction:
(30) $${\hat{y}} = {g}\left( {|{o}\rangle } \right).$$
  The predicted output ${\hat{y}}$ signifies the network’s prediction of disease status.

In AD detection, the loss function measures the difference between the expected outcomes and actual labels. Frequently used in binary classification applications, such as detecting AD, binary cross-entropy loss is employed. The loss $L$ is calculated for each data point $({x_i},{y_i})$, in which ${x_i}$ is the input data and ${y_i}$ is the corresponding label indicating the presence (1) or absence (0) of AD. The binary cross-entropy loss was formulated using the following formula:

(31) $$L\left( {{y_i},{{\hat y}_i}} \right) = - \left( {{y_i}.log\left( {{{\hat y}_i}} \right) + \left( {1 - {y_i}} \right).log\left( {1 - {{\hat y}_i}} \right)} \right).$$

Here, ${y_i}$ is the actual label (1 for Alzheimer’s, 0 for non-Alzheimer’s), ${\hat y_i}$ is the predicted probability of AD for input ${x_i}$. To compute the overall loss over the entire dataset, the individual losses were typically averaged:

(32) $$Loss = \displaystyle{1 \over N} \sum \limits_{i = 1}^N L\left( {{y_i},{{\hat y}_i}} \right),$$

where $N$ represents the total number of data points. The training objective is to reduce this loss as much as possible by modifying the model parameters using strategies such as gradient descent. This guarantees that the model is capable of diagnosing AD accurately and is enhanced by the tight match between its predictions and truth labels. The overall effect of the QDNN forward pass is that it uses quantum state encoding and parallel computation within cost-effective hybrid activation schemes to yield richer representations of features.

**Algorithm 3:** Pseudocode for simulated annealing in QDNN.

Input: QDNN model, input features
Output: Output prediction
Begin
Encode input features into quantum states.
For each layer in the QDNN model:
Apply quantum gates to the quantum states.
Measure quantum states to obtain classical values.
Encode classical values back into quantum states.
Apply quantum gates to the quantum states in the output layer.
Measure the final quantum state to obtain the output prediction.
Return the output prediction.
End


**Simulated Annealing in QDNN:** SA, or metallurgical annealing, is a probabilistic optimisation technique. It helps QDNN by better exploring the solution space, resolving possible issues with quantum entanglement, and identifying the best configurations for models that forecast diseases. It is employed to iteratively search the solution space for the global minimum of a function. The method mimics substance cooling gradually, where the likelihood of accepting a worse solution diminishes with time.

**Algorithm 4:** Simulated annealing optimisation for QDNN parameter tuning.

Input: QDNN parameters, initial temperature, cooling rate, maximum iterations
Output: Optimised QDNN parameters
Compute the present energy as the objective function value.
Set the best solution as the present solution.
Set the best energy as the present energy.
Set the temperature to the initial temperature.
For each iteration in the maximum iterations:
Generate a proposed solution by perturbing the present solution.
Compute the proposed energy.
Calculate the energy difference between the proposed and present energy.

Objective Function $E\left( x \right)$: Defining $E\left( x \right)$ corresponding to the loss function of the QDNN.

(33) $$E\left( x \right) = Loss\left( {QDNN\left( x \right)} \right).$$

Here, $x$ represents the QDNN parameters, $QDNN\left( x \right)$ is the QDNN model with parameter $x$, and loss $\left( . \right)$ is the loss function.

Temperature Schedule: SA uses a temperature schedule to regulate the likelihood of accepting uphill moves. The temperature begins high enough to permit exploration and then progressively drops.

(34) $${{T}_{new}} = {\alpha }.{{T}_{old}},$$

where α is the cooling rate.

Acceptance Probability: The QDNN parameters were updated in each iteration based on the Metropolis acceptance criterion. Assuming the present solution $x$, the possibility of granting a transition to a new solution $x'$ is as follows:

(35) $$P\left( {accept} \right) = \exp \left( { - \; \displaystyle{{E\left( {{x}^{\prime}} \right) - E\left( x \right)} \over T}} \right).$$

**Figure 2:**
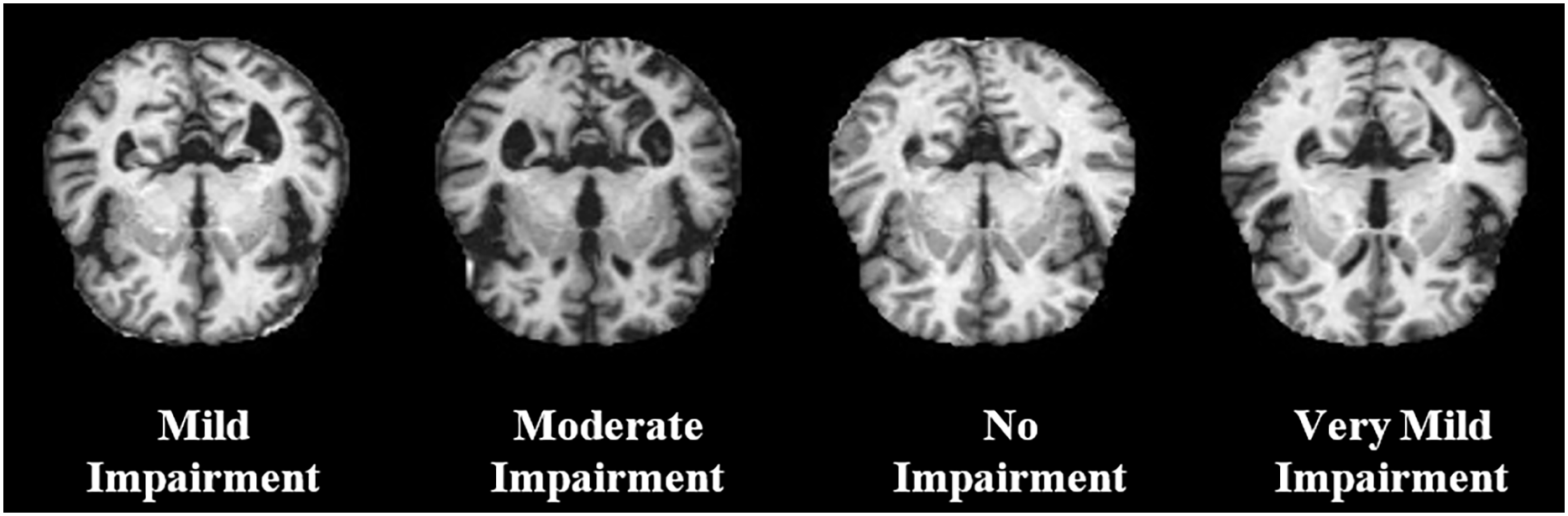
Detection by suggested model.

Here, $E\left( x \right)$ represents the current objective function value, $E\left( {{x}^{\prime}} \right)$ represents the proposed objective function value, and $T$ is the temperature parameter controlling the randomness of the search. The QDNN parameters were updated accordingly. This process is repeated until convergence. SA provides a robust optimisation approach for fine-tuning QDNN parameters in AD detection, ensuring that the model achieves optimal performance. The prediction of the AD by the suggested model was presented in the following Fig. 2, which contains mild impairment, no impairment, moderate impairment, and very mild impairment.

## Experimental result and discussion

The computational infrastructure was essential to implementing the proposed approach. Operating on Windows OS, the machine utilised a Core i9 CPU with a 5.8 GHz clock rate and 16 GB RAM. The hardware and software configuration selection is noteworthy because it illustrates the necessity of a stable computing environment to manage the intricacy of the AD detection model. Additionally, the study’s data came from the AD neuroimaging effort.

### Hyperparameter optimisation

Hyperparameter optimisation is critical to training ML models to ensure optimal performance. In AD detection using QDNNs with Haralick feature extraction and SA optimisation, tuning specific hyperparameters is essential for achieving accurate and efficient results, as shown in Table 2.

**Table 2:** Hyperparameters and their values.

Hyperparameter	Description	Values or range
Quantum learning rate	Rate at which the QDNN adapts to the data	0.01
Quantum epochs	Number of passes through the quantum network	200
Quantum batch size	Number of samples processed in each quantum batch	32
Haralick texture window size	Size of the window for Haralick feature extraction	9
Simulated annealing (SA) iterations	Number of iterations in the SA optimization	500
SA temperature	Initial temperature for SA	1.0
SA cooling	Cooling rate for temperature decay	0.95

**Figure 3:**
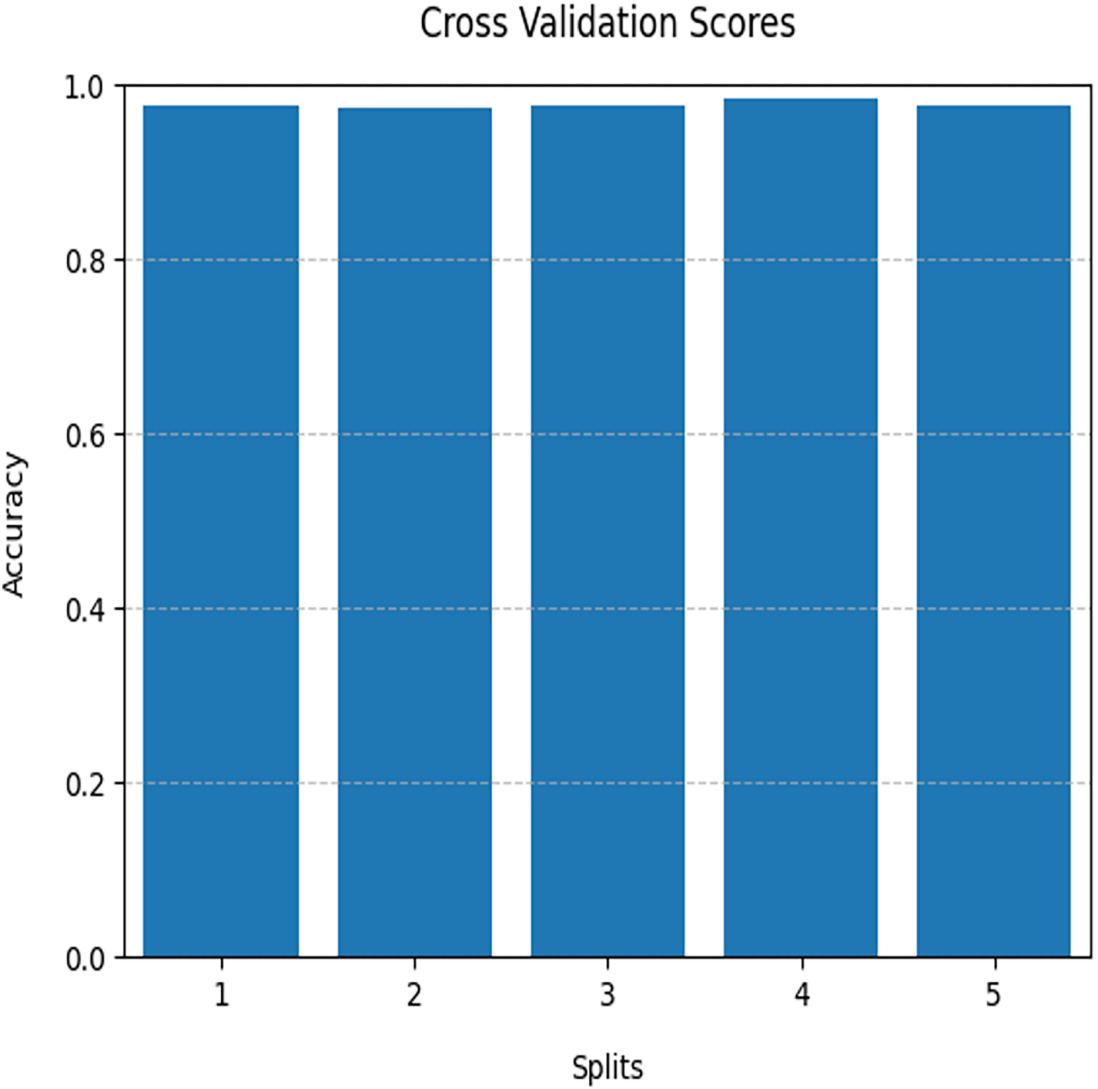
Plot of accuracy *vs*. different folds of cross validation for QDNN model.

The performance of the proposed model was evaluated on the Kaggle dataset, showing promising results in AD detection. The optimised QDNN, combined with Haralick features and SA, demonstrated enhanced accuracy, precision, sensitivity, and specificity compared to traditional methods. The performances of the various models are compared in Table 3. The proposed QDNN outperformed the other models across multiple metrics. The QDNN model’s 0.98 accuracy indicates its ability to classify instances properly. The FSSA used in this work dynamically adjusts the contribution of each Haralick feature, ensuring that the most informative and disease-specific patterns are prioritised while redundant or noisy features are minimised. In addition, the FSSA framework prevents overfitting by optimising the feature weights rather than relying on the entire feature set. The graph neural network (GNN) follows closely, with an accuracy of 0.90, showing its ability to leverage graph structures for effective information processing (Fig. 3). While traditional ML models such as XGBoost, multi-layer perceptron (MLP), and support vector machine (SVM) exhibit respectable accuracies ranging from 0.86 to 0.92, the proposed QDNN stands out as the optimal choice for the given task.

**Table 3:** Comparison of performance of various models.

Model name	Accuracy	Precision	Sensitivity	Specificity
Proposed QDNN	0.98	0.99	0.97	0.98
Graph neural network	0.90	0.87	0.89	0.88
XGBoost	0.88	0.84	0.87	0.85
MLP	0.86	0.81	0.84	0.82
SVM	0.92	0.91	0.88	0.89

The proposed model’s precision (0.99) and sensitivity (0.97) highlight its ability to identify true-positive cases and minimise false negatives accurately. The integration of FSSA in the QDNN model emphasises the most discriminative texture features, leading to more confident and precise positive classifications. The model accurately distinguished between patients and healthy controls by fine-tuning the Haralick features specific to AD pathology, reducing misclassification. While still respectable, the precision scores for the other models were comparatively lower, with GNN at 0.87, XGBoost at 0.84, MLP at 0.81, and SVM at 0.91.

The use of quantum computing principles in the QDNN model, coupled with optimised feature extraction techniques such as Haralick feature extraction and SA optimisation, likely contributes to its exceptional precision, showcasing the potential of quantum DL in advancing AD detection methodologies. The high sensitivity of the proposed model demonstrates its superior capability for correctly detecting cases of interest. The sensitivity of the proposed model to the concentration shows that it performs well in correctly detecting cases of interest. It is more sensitive than other models, such as GNN (0.89), XGBoost (0.87), MLP (0.84), and SVM (0.88), which implies a high potential impact on the early findings of AD.

In addition, the specificity of the QDNN demonstrated the functionality to correctly recognise true negative instances. Comparing the proposed results with other models such as GNN, XGBoost, MLP, and SVM shows that the proposed QDNN always produces larger values for all metrics. Because FSSA reduces the chance of false positives, the specificity of the QDNN model can be improved. The Proposed QDNN achieves an impressive specificity score of 0.98, outperforming all other models. This highlights the effectiveness of integrating quantum principles into DNNs for AD detection.

### Cross-validation

**Figure 4:**
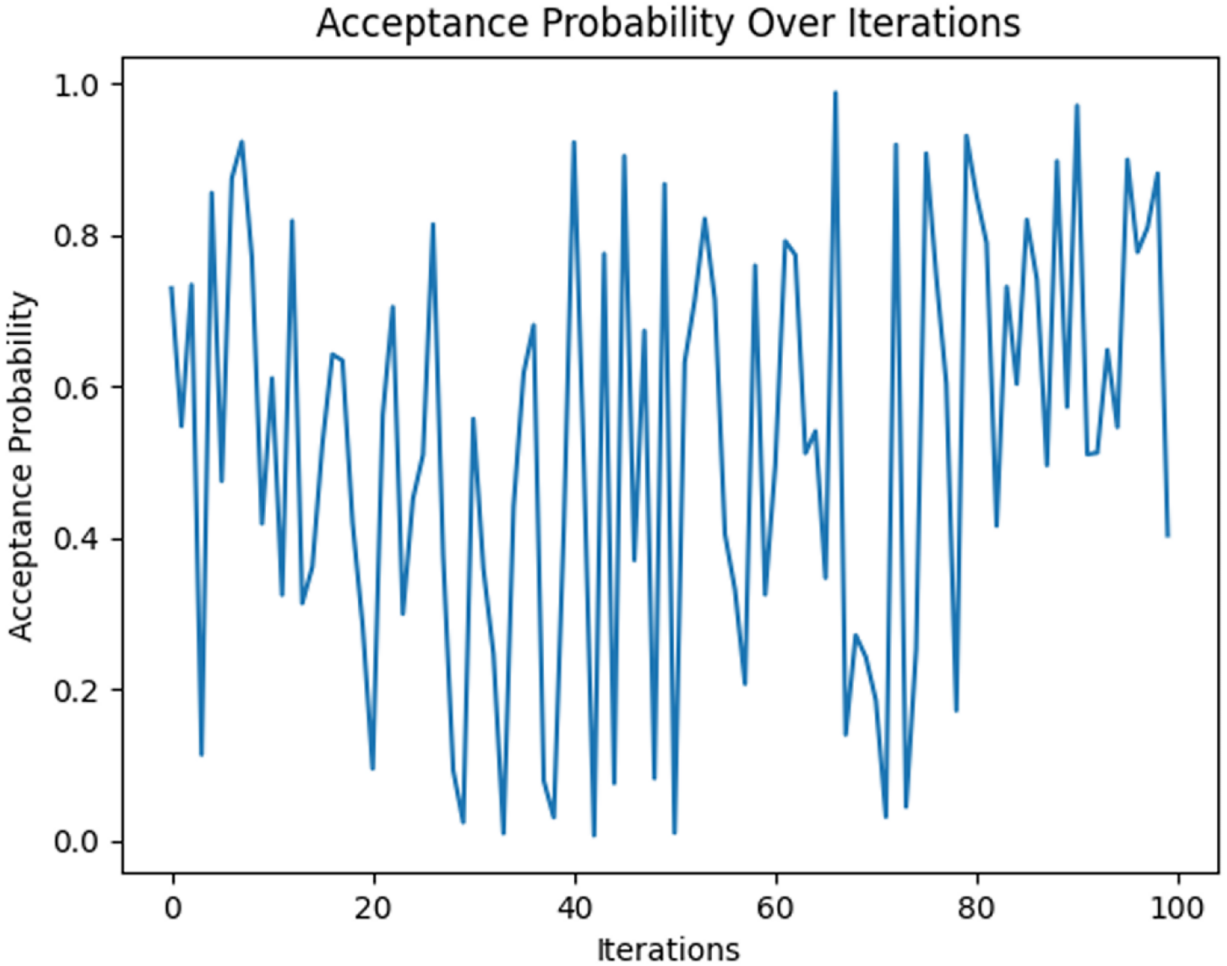
Acceptance probability over iterations for QDNN model.

Cross-validation, a model evaluation method, separates the original and training datasets. This method trains and evaluates the algorithm. The original dataset was partitioned into k subgroups of equal size. It was divided using k-fold cross-validation. The k-1 sets were identified as training data, while the remainder was utilised to validate the model. This algorithm was run k times (k represents the number of folds). Each subset is utilised once for the validation data. This algorithm scores the QDNN model, which yields variable levels of accuracy across different folds. It was assessed (averaged over numerous iterations) to assess the classification performance. Figure 4 illustrates the 98% detection accuracy of the QDNN model for AD. The acceptance probability remains relatively high at the beginning of the optimisation process. It is because the temperature was at its peak. It permits the algorithm to accept even worse solutions with a high likelihood. Finding the solution space broadly is important, thereby preventing premature convergence to local optima.

**Figure 5:**
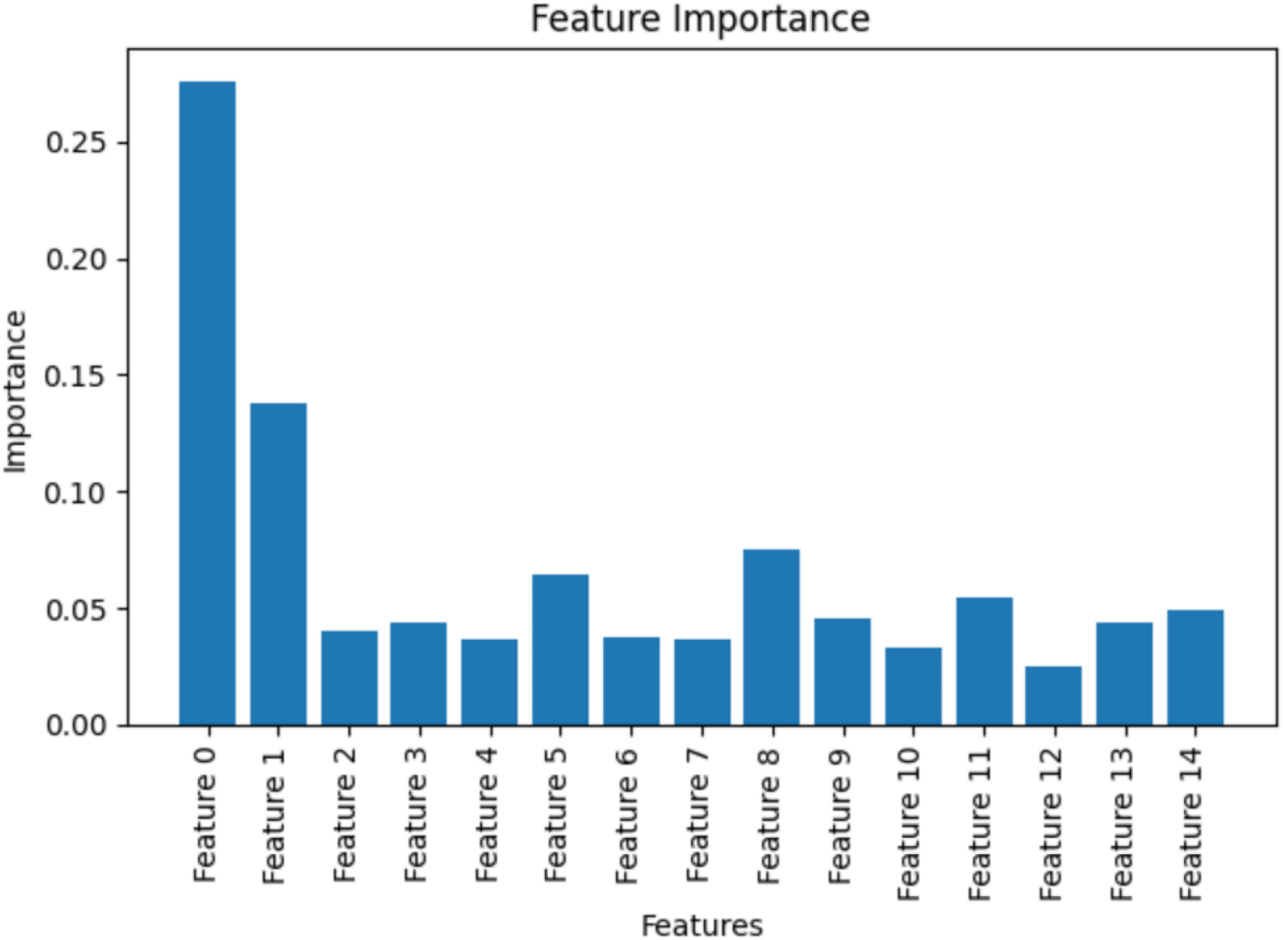
Feature importance plot for QDNN model.

**Figure 6:**
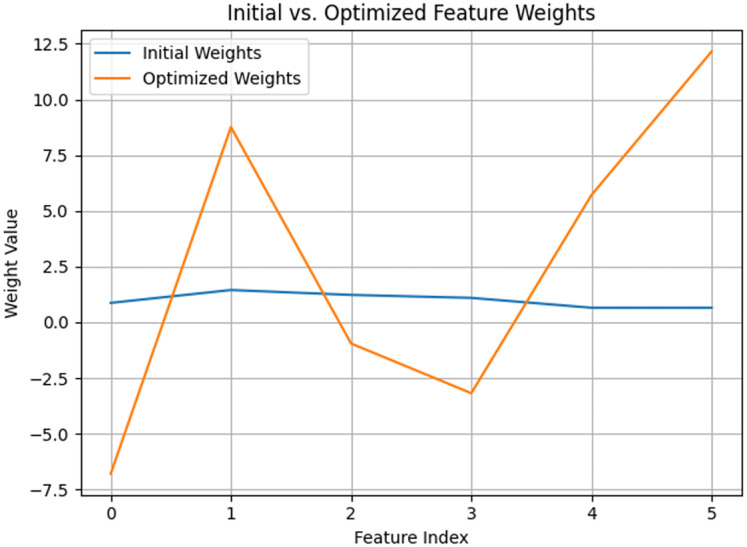
Initial *vs*. optimized feature weights in AD detection for QDNN model.

As iterations progressed (iteration = 100), the temperature slowly decreased according to the cooling schedule. This will reduce the acceptance probability, which is shown in Fig. 5. This controlled reduction enhances exploitation by refining the solution and stabilising the parameters of the model. During this phase, the FSSA method strategically adjusts the feature weights of the Haralick texture parameters, ensuring the retention of only the most informative features relevant to Alzheimer’s detection. The feature importance plot highlights different Haralick features such as correlation, contrast, homogeneity, energy, entropy, and dissimilarity to the QDNN model’s predictive performance (Fig. 6). Haralick features are extracted for different angles ([0, np.pi/4, np.pi/2, 3*np.pi/4]), & distances ([1, 3, 5, 7]). Features (0, 1, 8), such as contrast, entropy, and correlation, exhibit the highest importance scores. These features capture critical textural variations that reflect neurodegenerative changes in brain images.

Specifically, contrast measures local intensity differences and plays a key role in detecting structural abnormalities associated with AD. Entropy reflects the complexity of image patterns and is often increased in pathological brain regions. Correlation captures the linear dependency between neighbouring pixel intensities, providing insight into tissue homogeneity loss due to neurodegeneration. Features such as homogeneity and energy show moderate importance. While these features are informative, their contribution is slightly lower owing to their sensitivity to smooth and regular patterns, which are less discriminative in later Alzheimer’s stages. Certain redundant or noisy features, including dissimilarity, are assigned lower weights through FSSA optimisation. It demonstrates the model’s ability to downweight features that provide limited new information, improving efficiency without compromising accuracy.

**Figure 7:**
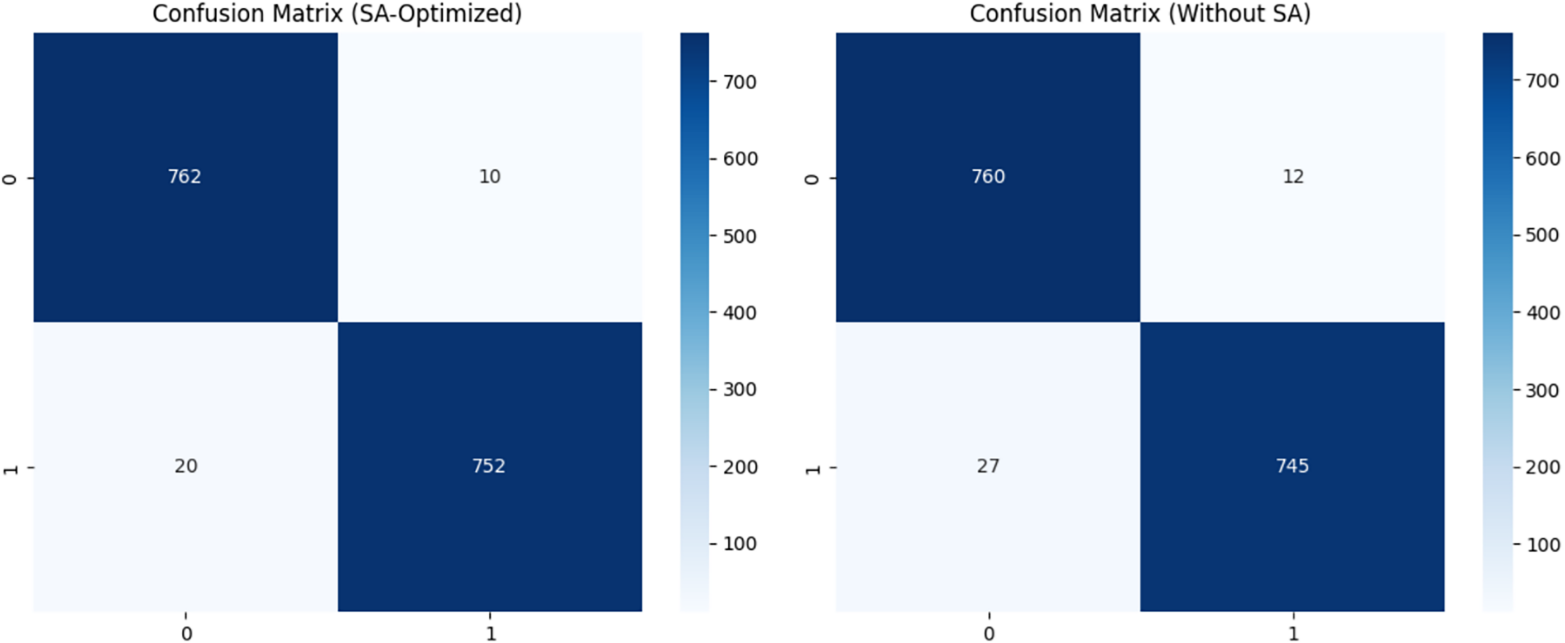
Confusion matrix with FSSA-optimized and without FSSA for QDNN model.

In the initial feature weight distribution, the QDNN model (Fig. 7) exhibited a uniform allocation across all Haralick features, resulting in minimal differentiation between high- and low-importance features. This lack of distinction reduces the model’s ability to capture subtle structural changes associated with early-stage AD, limiting its sensitivity to critical diagnostic patterns. In contrast, the optimised feature weights obtained through the FSSA approach demonstrated a more refined and adaptive distribution. Notably, the weights for contrast and correlation increased because these features were particularly effective at capturing intensity variations and spatial dependencies reflective of tissue degradation.

Fewer significant features received lower weights, thereby minimising their influence and reducing noise in the model. Furthermore, the FSSA method facilitates dynamic adjustments of feature importance based on real-time model feedback during optimisation. It prioritises the most informative features, improving accuracy and AD detection efficiency. A confusion matrix table is used to assess the efficacy of the classification model, which will provide all the details related to the model’s predictions along with data classification. True positive (TP) refers to the number of samples identified adequately as positive. True negative (TN) signifies the number of samples accurately grouped as negative. In addition, false positive (FP) denotes the instances erroneously labelled as positive. Finally, false negative (FN) indicates cases inaccurately classified as negative by the model.

**Figure 8:**
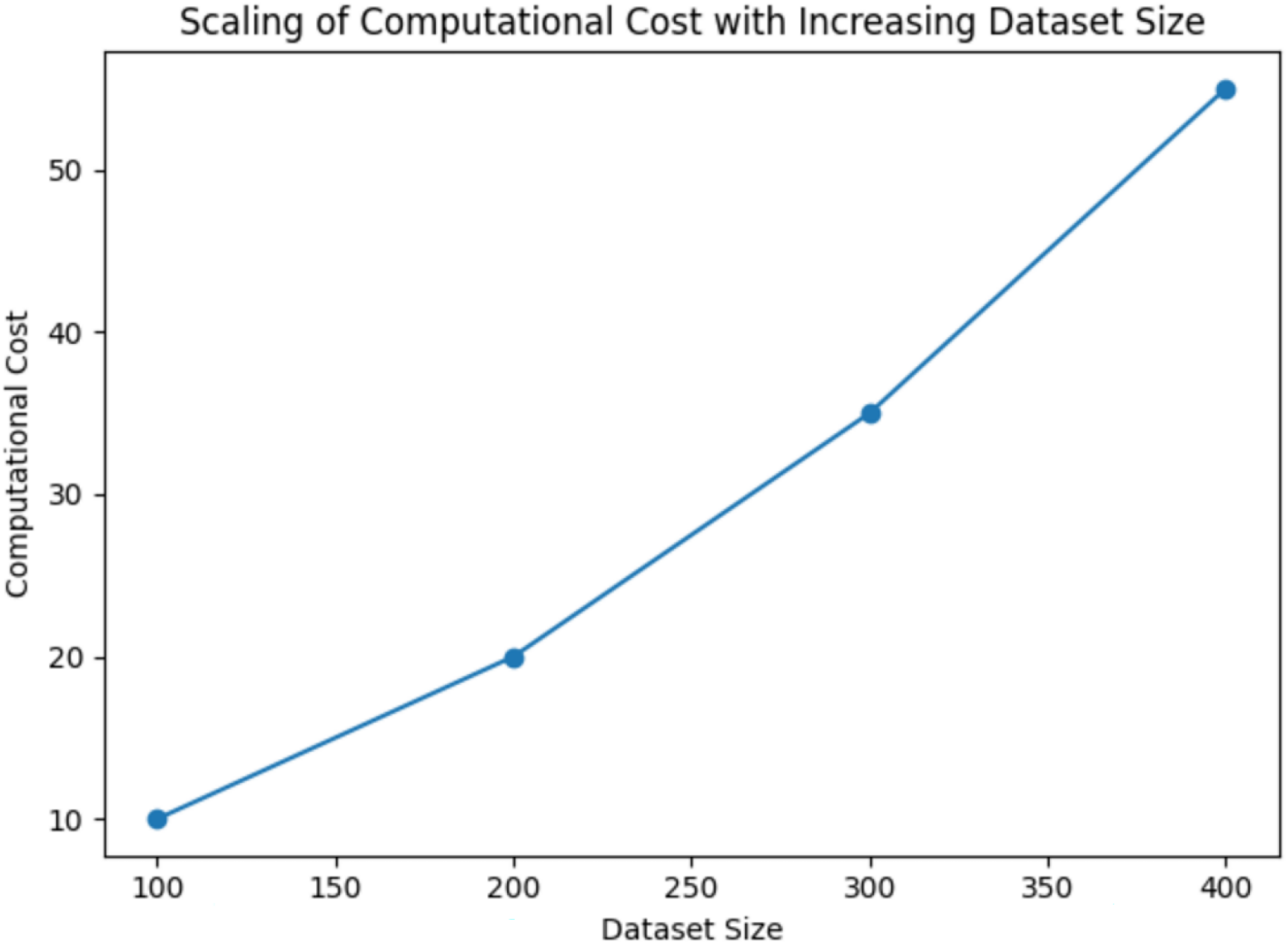
Scaling of computational cost with increasing dataset size for QDNN model.

The confusion matrix reveals a notable improvement in overall accuracy when incorporating FSSA into the optimisation process, as depicted in Fig. 8. Without FSSA, the model showed more misclassified samples, particularly in borderline cases with subtle or ambiguous texture features. With FSSA, the model optimises the weights of critical Haralick features, allowing a better distinction between healthy and Alzheimer-affected patients. Without FSSA, a higher FN indicates that the QDNN model struggles to detect the early stages of AD. With FSSA, enhanced TP and TN rates indicate that feature-specific optimisation effectively improves the ability of the model to find the difference between classes. The improved accuracy highlights FSSA’s ability to prioritise diagnostically significant Haralick features, thereby minimising the influence of redundant or noisy features.

**Figure 9:**
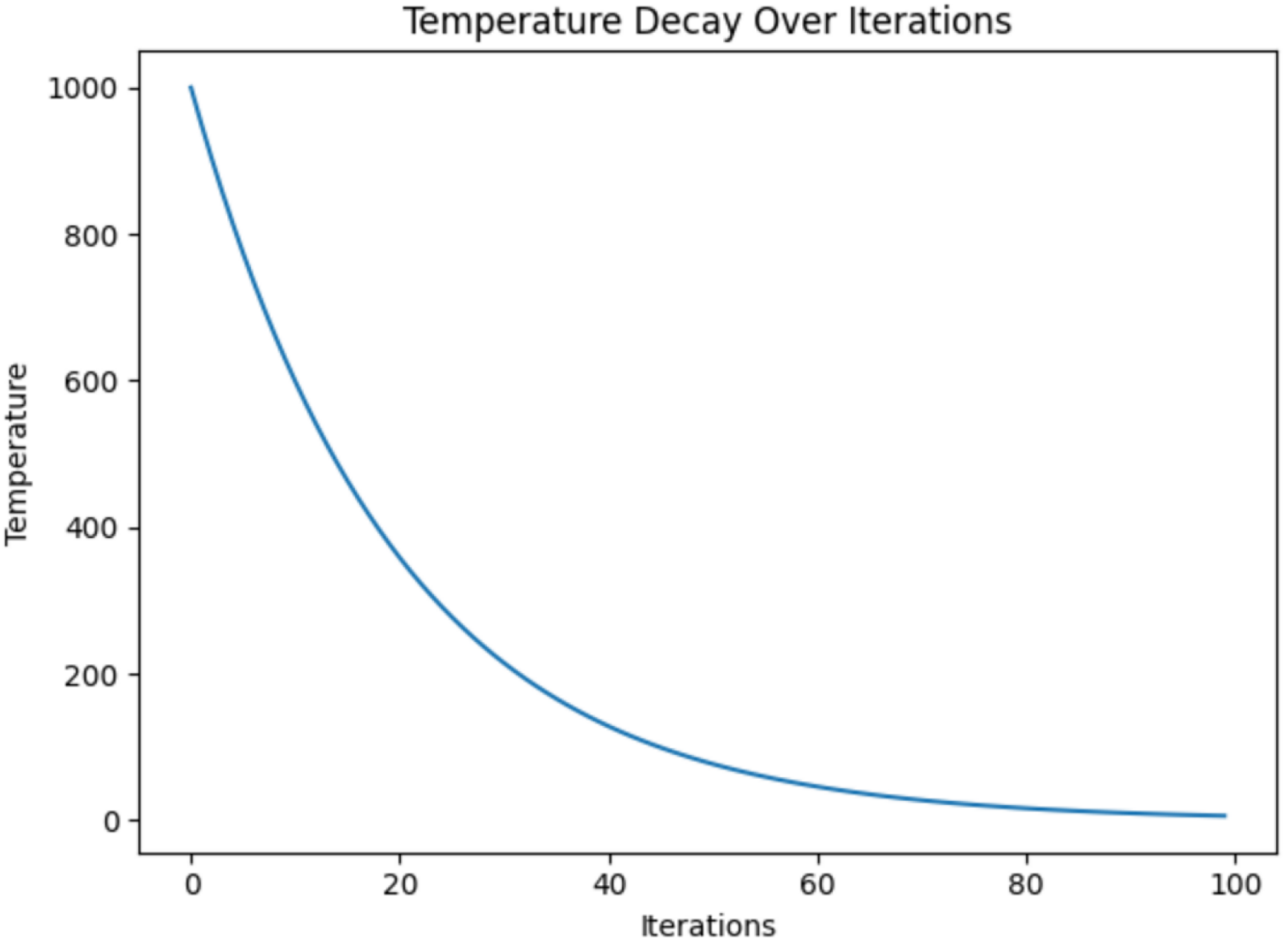
Temperature decay over iterations in FSSA.

Figure 9 indicates that FSSA scales sub-linearly with increasing dataset size due to the optimisation space’s reduced dimensionality. As the dataset size increases, the QDNN model mitigates the computational overhead through FSSA and quantum-enhanced computation. This innovation improves diagnostic accuracy and ensures that the framework remains computationally feasible for large-scale medical datasets, making it a promising solution for real-world AD detection.

**Figure 10:**
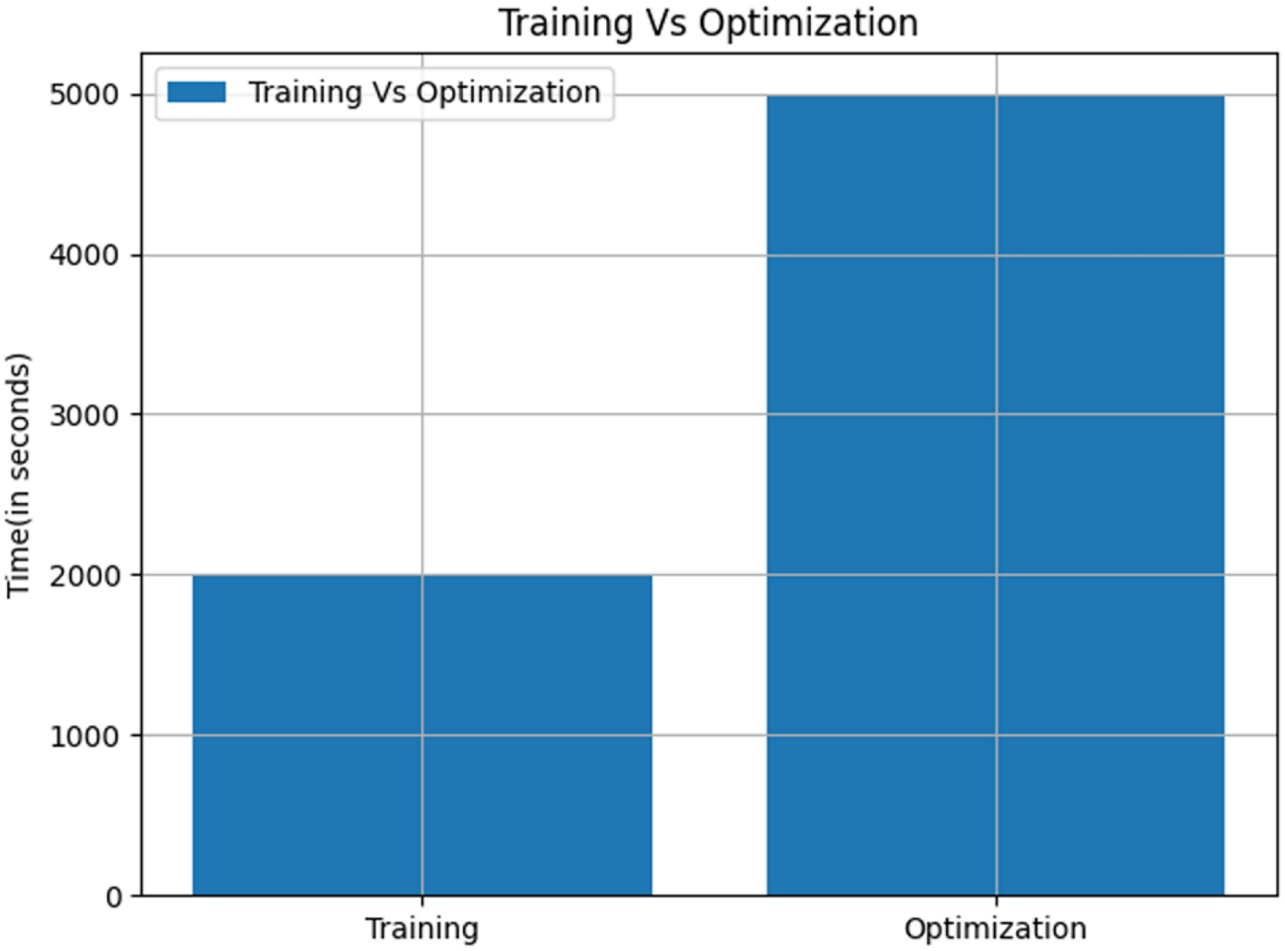
Training time *vs* optimization time for QDNN model.

A critical observation in the proposed QDNN model is the balance between reducing the training time and maintaining a reasonable optimisation time. FSSA significantly reduces the complexity of the feature set before training, leading to faster model convergence. Although the optimisation phase requires additional computational steps, the reduction in feature redundancy compensates for this overhead. It is illustrated in Fig. 10. Table 4 presents the performance comparison in the field of AD.

**Table 4:** Performance analysis of different methods in AD on same publicly available dataset.

Models	Accuracy
Multiple instance learning (Tong et al., 2014)	90
3D CNN for Early AD Detection (Ramani et al., 2024)	95
EfficientNet-B0 (Ching, Abdullah & Shapiai, 2024)	87.17
Multiple ML models (Baglat et al., 2022)	86
Proposed	98

A comparative ablation study was performed to demonstrate the success of the proposed model and compare it to existing AD detection methods applied to the task using the same publicly available dataset (Table 4). The accuracy rate of the proposed QDNN is better (98%), which surpasses all other models that were compared. Particularly, it had 8% growth relative to multiple instance learning (Tong et al., 2014) of 90% and 3% growth relative to 3D CNN for early AD detection (Ramani et al., 2024) of 95% as well as 10.83% growth relative to EfficientNet-B0 (Ching, Abdullah & Shapiai, 2024) whose accuracy was at 87.17%. Additionally, the proposed model produced better results than the multiple ML models (Baglat et al., 2022) with 86% that have limitations due to lack of powerful hybrid or deep artificial intelligence (AI) architecture. Although EfficientNet-B0 was not very effective in capturing complex information related to AD characteristics because it uses generic image representations that are trained in advance, the proposed QDNN managed to address this limitation by embedding Haralick texture feature extraction to encode rich structural properties and textures on the MRI images, FSSA to remove redundancy and increase the discriminative ability as well as using quantum deep learning to represent sophisticated feature dependencies. This synergetic combination greatly expanded the ability of the model to detect small patterns that signal AD, resulting in super-performance. Table 5 shows the performance analysis on different datasets.

**Table 5:** Performance analysis on different datasets.

Dataset	Precision	F1-score
ICHDR (Periasamy et al., 2023)	98	95
HMS (Assam et al., 2021)	96	98
ADNI (Chu & Gebre-Amlak, 2021)	97	97

A comparative testing performed on various publicly available datasets indicates the scale and flexibility of the proposed model in different data-set characteristics and complexities. In the ICHDR dataset (Periasamy et al., 2023), the model had a precision of 98%, and F1-score of 95% implying excellent accurate predictions of positive results and good balance between precision and recall. In the case of the HMS dataset (Assam et al., 2021), the model reported a precision of 96 and an F1-score of 98, implying a better ability to find relevant instances also in varying clinical imaging scenarios. The performance remained high, with a precision of 97% and F1-score of 97% based on the ADNI dataset (Chu & Gebre-Amlak, 2021), demonstrating the consistency and stability of the model regarding the various MRI sources. These findings validate the presented method as generalizable with robust predictive ability regardless of dataset-specific imaging variance.

**Figure 11:**
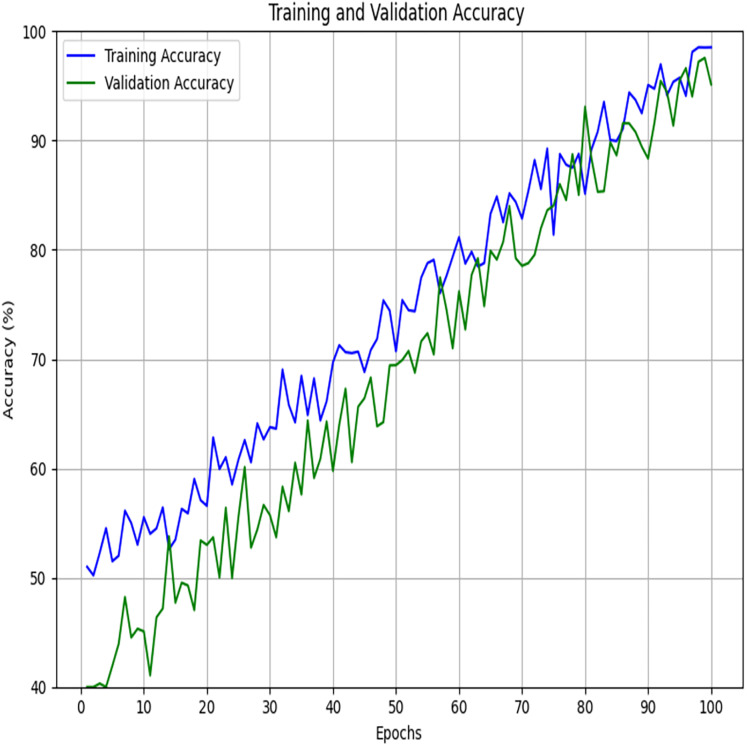
Training and validation accuracy.

**Figure 12:**
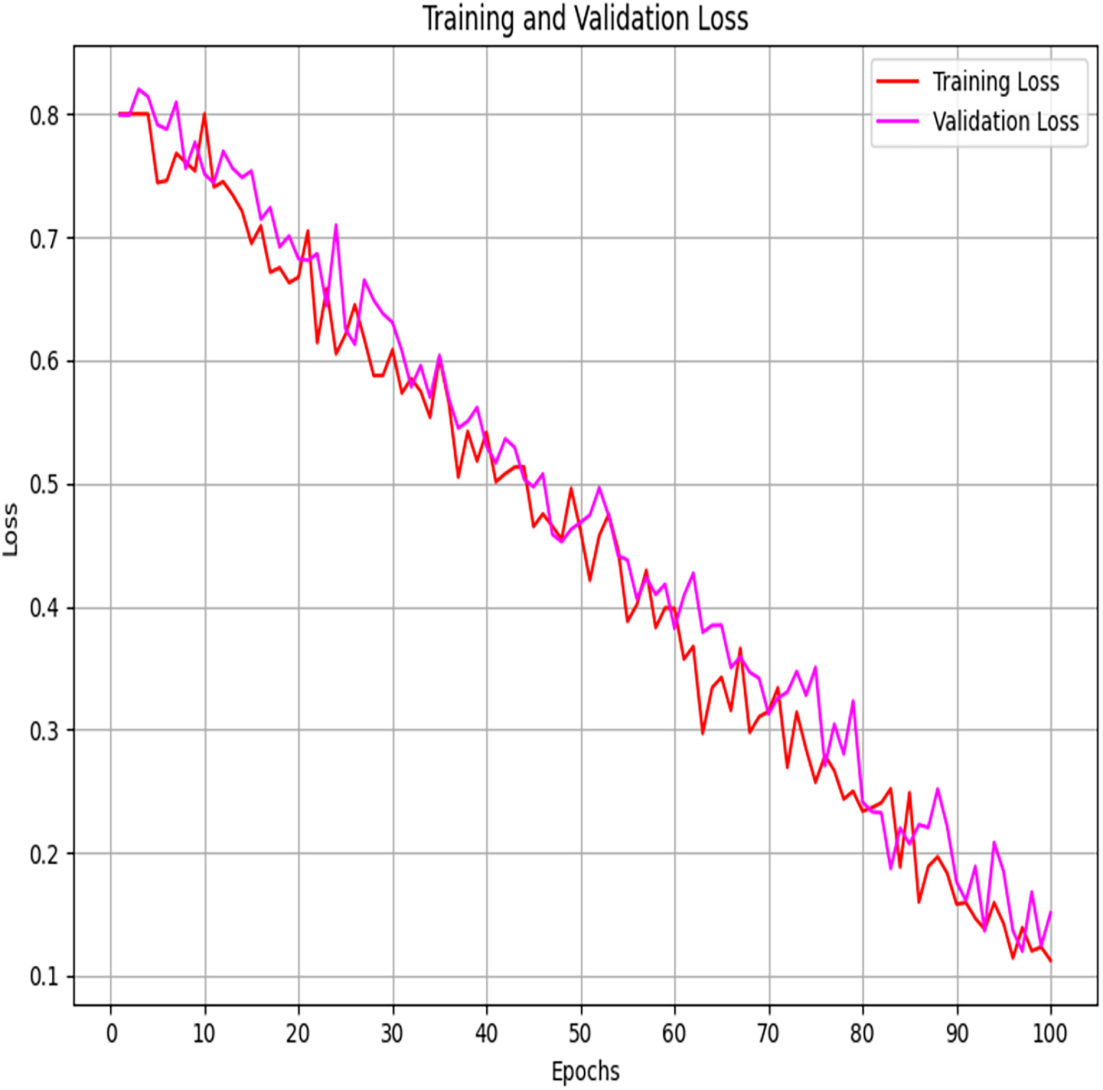
Training and validation loss.

The plots of accuracy and loss of training and validation (Figs. 11 and 12) clearly illustrate the convergence and generalisation potential of the suggested model. As illustrated, training accuracy progressively improves with improvement by roughly 50% to 98%, whereas validation accuracy has a parallel but slightly smaller improvement trajectory, denoting that the model learns useful discriminative features without large variation between training accuracy and validation accuracy. Relatedly, training loss and validation loss are consistently reducing by approximately 0.8 up to 0.1, without any sharp deviations, indicating a low level of overfitting. This accuracy and low loss can be explained by combining the FSSA approach to optimal feature selection and optimizing the parameters of QDNN so that they increase the relevance of features and model flexibility. Accuracy and loss curves are closely similar across epochs, indicative of a well-regularized model design and good parameter search resulting in stable convergence and biasness-variance trade-off. These findings support that the suggested model can attain high learning stability and preservation of generalization to unseen data.

An ablation study was conducted to show the individual role of each component used in the proposed model architecture in AD detection. The baseline QDNN model has been used, it has been trained on preprocessed data, and no further features were extracted or optimized. This benchmark acted as a point of reference to determine the enhancement brought about by later elements. In the second form, Haralick feature extraction was incorporated in order to extract fine-grained texture patterns in the medical images which are important in detecting small abnormalities caused by AD. And this addition lead to significant increase in the classification performance and it can be seen that inclusion of such handcrafted texture descriptors has improved the performance of the model to differentiate between stages of AD. Lastly, adding FSSA even enhanced performance to 98%, showing it could optimize weighting of features to emphasize most discriminative patterns and inhibit redundant or noiseful ones. The results, which were obtained after the integration of FSSA with Haralick features and QDNN achieved the highest accuracy, and the smallest loss guaranteed the optimization step not only minimized the redundancy, but also enhanced convergence during the training. The step-by-step assessment demonstrates the complementary nature of feature extraction and optimization in the proposed architecture, as it is each module of the architecture that increases the robustness, precision, and generalization ability of the final AD detection framework.

## Conclusion

This study proposed a novel framework for AD detection by constructing QDNNs using Haralick texture feature extraction and SA-based optimisation. A better classification accuracy could be achieved on the proposed QDNN model through FSSA optimisation than other traditional machine learning models, including GNN, XGBoost, MLP, and SVM. Haralick features integration improved the model’s ability to capture microtextural changes. The FSSA method effectively prioritised features, reduced overfitting, and increased sensitivity and specificity. The robustness and generalisability of the model were identified through cross-validation on several folds. The convergence and optimisation of feature weights were also mitigated through the adaptive temperature decay mechanism of the FSSA. These findings are generally consistent with potential improvements in the early stages of AD detection with quantum-inspired DL frameworks. This study not only provides a solution to the problem of achieving accurate and fast medical image classification but also opens the path to developing scalable quantum-integrated diagnostic tools in clinical neuroscience.

## Supplemental Information

10.7717/peerj-cs.3387/supp-1 Supplemental Information 1 Source Code of Alzheimer disease detection using quantum deep neural network.

## Additional Information and Declarations

### Data Availability

The following information was supplied regarding data availability:

The data is available at Kaggle: https://www.kaggle.com/datasets/lukechugh/best-alzheimer-mri-dataset-99-accuracy.

The source code is available in the Supplemental File.
